# Vitamin D—A New Therapeutic Target in the Management of Type 2 Diabetes Patients

**DOI:** 10.3390/jcm13051390

**Published:** 2024-02-28

**Authors:** Oana Albai, Adina Braha, Bogdan Timar, Ioana Golu, Romulus Timar

**Affiliations:** 1 Department of Second Internal Medicine—Diabetes, Nutrition, Metabolic Diseases, and Systemic Rheumatology, “Victor Babes” University of Medicine and Pharmacy, 300041 Timisoara, Romania; albai.oana@umft.ro (O.A.); bogdan.timar@umft.ro (B.T.); timar.romulus@umft.ro (R.T.); 2 Department of Diabetes, Nutrition and Metabolic Diseases Clinic, “Pius Brînzeu” Emergency Clinical County University Hospital, 300723 Timisoara, Romania; 3 Centre for Molecular Research in Nephrology and Vascular Disease/MOL-NEPHRO-VASC, “Victor Babes” University of Medicine and Pharmacy, 300041 Timisoara, Romania; 4 Department of Endocrinology, “Victor Babes” University of Medicine and Pharmacy, 300041 Timisoara, Romania; golu.ioana@umft.ro

**Keywords:** vitamin D deficiency, type 2 diabetes mellitus, glycemic control, diabetic polyneuropathy, neoplasia, cardiovascular disease, dementia, stroke

## Abstract

**Background**: Vitamin D is a fat-soluble vitamin that prevents cardiovascular diseases and diabetes mellitus (DM). The present research aimed to study the impact of 25-hydroxyvitamin D (25(OH)D) level on the health status of patients with type 2 DM (T2DM) hospitalized in the “Pius Brînzeu” Emergency Clinical County University Hospital in Timisoara, Romania. **Methods**: The study retrospectively included 160 patients with T2DM who were clinically and biologically evaluated during hospitalization. **Results**: 13.1% of patients had optimal, 23.1% insufficient, and 63.8% deficient 25(OH)D values. Patients with 25(OH)D deficiency presented poorer glycemic control and were older, with higher weight, but had altered renal function, anemia, and lower iron values. Also, patients with associated neoplasia, diabetic neuropathy, cardiovascular disease (CVD), dementia, and grade 3 arterial hypertension (HTN) had lower values of 25(OH)D. An age > 55 years (sensitivity 69.9, specificity 82.5, AUROC 0.786, *p <* 0.001) and an HbA1c > 7.7% (sensitivity 89.3, specificity 92.9, AUROC 0.938, *p <* 0.001) predict 25(OH)D deficiency in T2DM patients. **Conclusions**: Vitamin D influences almost every system and organ in the body, so it should be a routine test for all patients with DM to correct the deficiency and prevent other diseases and complications.

## 1. Introduction

Diabetes mellitus (DM) is a major and real problem for health systems worldwide. Its prevalence has been increasing alarmingly in recent years: it is currently estimated that 537 million adults (aged 20 to 79) have DM, which is expected to increase to 643 million in 2030 and to 783 million in 2045 [1].

There is also an increasing prevalence of vitamin D deficiency. Vitamin D, considered the sunshine vitamin, is a liposoluble vitamin naturally present in very few foods and produced in the skin under the effect of ultraviolet rays (UV). Food sources of vitamin D are fatty fish and fish liver oil (cod). However, cod liver oil is also an important source of vitamin A, and consuming it in large quantities increases the risk of vitamin A poisoning [2]. Other food sources that naturally contain vitamin D are mushrooms, egg yolk, and liver. Moreover, some products are artificially enriched with vitamin D (breakfast cereals, orange juice, and some milk formulas) [3]. The main source of vitamin D is the skin [4], the synthesis of vitamin D in the skin being dependent on a series of geographical factors (latitude, season, degree of sunshine, and pollution) and personal factors (time spent outdoors, age, body composition, degree skin pigmentation, genetic factors) [5]. Depending on the source, vitamin D (calciferol) is found in the form of ergocalciferol (vitamin D2), synthesized especially in plants under the action of UV light, and cholecalciferol (vitamin D3), synthesized in the skin (in the deep layer of the epidermis and dermis). Vitamin D obtained from exposure to sunlight, food, and supplements is then transformed through two hydroxylation reactions. The first occurs in the liver and transforms vitamin D into 25-hydroxyvitamin D [25(OH)D], also known as calcidiol. The second occurs primarily in the kidneys and forms the biologically active 1,25-dihydroxyvitamin D [1,25(OH)2D], also known as calcitriol [3,6].

Most of vitamin D’s actions appear to be mediated by a nuclear transcription factor (VDR). After entering the nucleus, 1-25-dihydroxyvitamin D binds to the VDR and recruits another nuclear receptor, the retinoid X receptor (RXR). In the presence of 1-25-dihydroxy vitamin D, the VDR/RXR complex binds small DNA sequences called vitamin D response elements (VDREs). It initiates a cascade of molecular interactions that modulate the transcription of specific genes. In the genome, thousands of VDREs have been identified. Also, the activation of VDR by 1-25-dihydroxyvitamin D seems to act directly or indirectly in the activity of approximately 100–1250 genes [7,8].

Vitamin D’s role is not limited to phosphor–calcium metabolism; it also has anti-inflammatory, anti-proliferative, and anti-infectious roles. The major role of vitamin D in its active form, 1,25(OH)2D3, is represented by the stimulation of the active absorption of calcium, which is necessary for bone mineralization. In addition, increasing plasma calcium to the normal value is also necessary for the functioning of the neuromuscular junction, vasodilatation, nerve transmission, and hormonal secretion. The vascular system has specific receptors on several of its cells: vascular smooth muscle cells, endothelial cells, and cardiomyocytes. These produce 1-alpha hydroxylase, which binds vitamin D and converts 25-hydroxyvitamin D to calcitriol. Calcitriol prevents the proliferation of smooth muscle cells, has anti-inflammatory properties, reduces coagulation, and controls the renin–angiotensin system [9,10]. Vitamin D deficiency can lead to various symptoms, including asthenia, fatigue, loss of bone density, muscle aches, and damage to the immune system [11].

Vitamin D deficiency appears to be linked to the induction and progression of DM, with a clear relationship between vitamin D, insulin secretion, insulin tolerance, and pancreatic beta-cell dysfunction. Glucose homeostasis is influenced by vitamin D, evidenced by the presence of VDR in pancreatic beta-cells and skeletal myocytes [12,13]. It has also been observed that persons living with obesity are deficient in vitamin D, and the explanation could be volumetric dilution in those with a high body mass index (BMI). The same amount of serum vitamin D, existing in people with normal weight, is diluted in people living with obesity because the vitamin is divided between different compartments (muscle, adipose, liver), which are higher in those with increased weight [14,15,16].

The study mainly aimed to evaluate vitamin D levels in patients with T2DM and their impact on health status. We analyzed possible predictive factors for vitamin D deficiency like glycemic control (fasting glycemia, postprandial glycemia, HbA1c), DM complications, associated comorbidities like cancer, dementia, and other metabolic and anthropometric parameters.

## 2. Materials and Methods

### 2.1. Study Design and Patients

In a retrospective observational study, we included patients with T2DM hospitalized in “Pius Brînzeu” Emergency Clinical County University Hospital in Timisoara from September 2023 to January 2024 for different medical reasons. This research was conducted under the Declaration of Helsinki (2013 version). The informed written consent was waived for retrospective study design, and patient data were anonymized. The Ethical Committee of the Emergency County Hospital Timisoara approved our study protocol (416/15 November 2023).

We identified 645 patients with DM who were hospitalized during the study period. Patients were included in the study based on the following criteria: age ≥ 18 years, body mass index (BMI) ≥ 18 kg/m^2^, Hb ≥ 7 g/dL, previously known diagnosis of T2DM, who had dosed the vitamin D during hospitalization. Exclusion criteria were: age < 18 years, BMI < 18 kg/m^2^, severe form of anemia (Hb < 7 g/dL), type 1 DM, those who received vitamin D supplements prior to the hospitalization for at least three months, hyperparathyroidism and liver cirrhosis (Child–Pugh C), missing data about the level of vitamin D.

### 2.2. Patients Medical Assessments

The following parameters were analyzed: Age, sex, weight, BMI, data on smoking and alcohol consumption, estimated glomerular filtration rate (eGFR), iron, hemoglobin, thyroid-stimulating hormone (TSH), glycated hemoglobin (HbA1c), fasting and postprandial glycemia, and fasting serum 25-hydroxyvitamin D (25(OH)D).

According to the Institute of Medicine and the World Health Organization, the optimal 25(OH)D is at least 30 ng/mL; values between 20–29 ng/mL are considered a suboptimal level, and <20 ng/mL means vitamin D deficiency [17,18,19]. Vitamin D intoxication is usually observed in values of 25(OH)D > 150 ng/mL [20,21].

Also, we evaluated the presence of associated comorbidities like arterial hypertension, neoplasms, dementia, and DM chronic complications: microvascular diseases (diabetic retinopathy, chronic kidney disease), macrovascular diseases (stroke, ischemic heart disease, diabetic arteriopathy), and neuropathy.

### 2.3. Statistical Analysis

Statistical analysis was performed using MedCalc^®^ Statistical Software version 22.016 (MedCalc Software Ltd., Ostend, Belgium; https://www.medcalc.org; 4 December 2023). The continuous variables were tested for normal distribution with the Shapiro–Wilk test and presented as mean and standard deviation if normally distributed, or median with minimum and maximum values, respectively average rank, if nonparametric. Categorical variables were presented as absolute values and percentages. The statistical characteristics of the frequency distributions were illustrated by violin-plot diagrams and described as five values: the minimum value, IQR (the first quartile (or lower quartile), the median, the third quartile), the maximum value, and the density plot.

To evaluate the differences between the indicators of the central tendency between the groups, we used unpaired Student’s *t*-test for comparing the arithmetic means of the parametric variables between two groups, Mann–Whitney-U tests for comparing two medians, ANOVA test to study the variation of the mean values between at least two groups, and Kruskal–Wallis test to study the variation of the median values between more than two groups. To test the statistical significance of the differences between percentages/proportions/prevalences, we used chi-square tests (for comparison of two proportions) and chi-square for trend (for comparison between more than two proportions).

The strength and direction of the associations between the numerical variables analyzed were evaluated with the help of correlation coefficients and correlograms. Positive correlations are displayed in blue, and negative correlations are in red. The intensity of colors indicates the power of correlation. In the case of bivariate regressions, we calculated the coefficient of determination (R^2^) to verify in what proportion the independent variable’s variation generates the dependent variable’s variation. In order to study the impact of numerical and continuous variables on 25(OH)D, we built univariate and multivariate linear and logistic regression models. With the help of Nagelkerke’s pseudo-coefficient of determination, we showed how the regression model built explains the occurrence of the dichotomous event (25(OH)D deficiency).

For evaluation of the predictive power of 25(OH)D deficiency based on a value of a continuous variable, we performed “Receiver-Operating Characteristics” analyses. Predictive performance is described through sensitivity and specificity. The optimal threshold value of the predictor was considered equal to the Youden index. To analyze the statistical significance of the predictive capacity, we compared the area under the ROC curves of the model created with the non-discriminant one (the area under the ROC curve = 0.5).

In the study, we calculated the 95% confidence interval and considered a *p*-value of less than 0.05 as significant for the statistical analyses.

## 3. Results

After applying the inclusion/exclusion criteria, the study group comprised 160 patients with T2DM, 50.6% men (81/160) with a median age of 57 [37; 69] years and diabetes duration of 9.5 [5; 15] years. The median 25(OH)D level was 17.3 [14.5; 27] ng/mL. Only 30.6% of patients (49/160) presented related symptoms for suboptimal levels of 25(OH)D, the most common being fatigue muscle and joint pain. The symptomatology was more expressed in patients with very low values of 25(OH)D (<10 ng/mL). There were no statistically significant differences between levels of 25(OH)D depending on smoking status or alcohol consumption. The main characteristics of the patients included in the study, compared by gender, are shown in Table 1 and compared with 25(OH)D levels in Table 2.

A total of 13.1% (21/160) patients had optimal 25(OH)D values, 23.1% (37/160) insufficiency, and 63.8% (102/160) deficiency. Patients with 25(OH)D deficiency presented with significantly higher values of HbA1c, both fasting and postprandial glycemia, were older, with higher weight and BMI, but had lower values of eGFR, Hb, and iron (Table 2).

**Figure 1:**
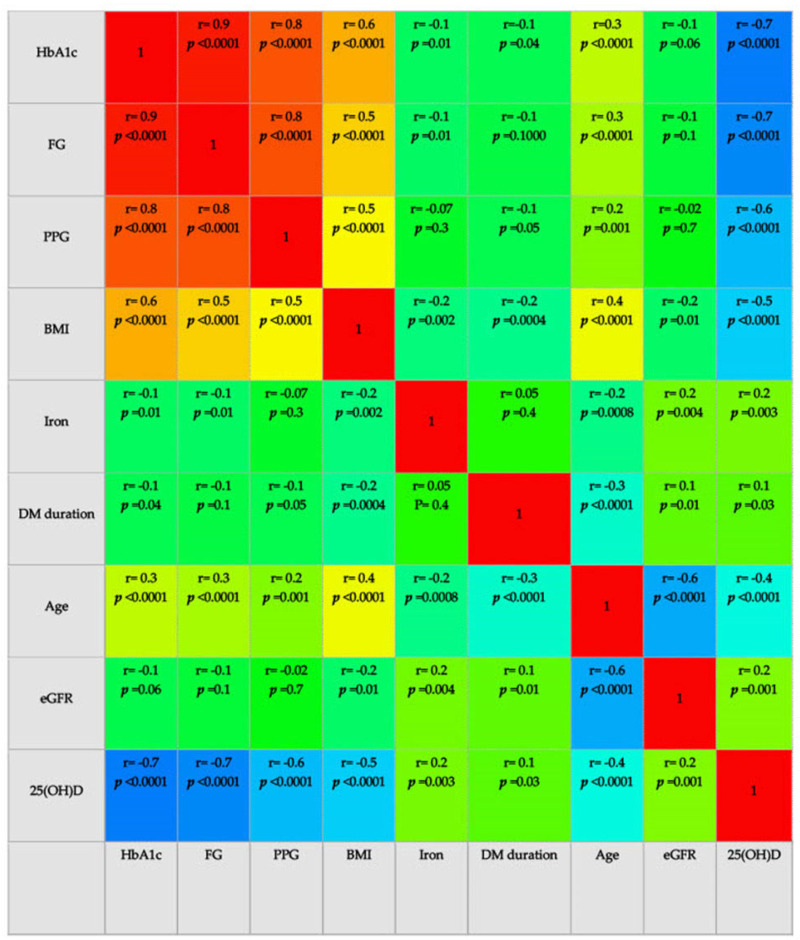
Correlogram of 25(OH)D and studied variables. Pearson correlation coefficient: Positive correlations are displayed in blue, and negative correlations are in red. The intensity of colors indicates the power of correlation. HbA1c = glycated hemoglobin; FG = fasting glycemia; PPG = postprandial glycemia; DM = diabetes mellitus; eGFR = estimated glomerular filtration rate; 25(OH)D = fasting serum 25-hydroxyvitamin D; *p <* 0.05 statistically significant. MedCalc^®^ Statistical Software version 22.016 (MedCalc Software Ltd., Ostend, Belgium; https://www.medcalc.org; 4 December 2023).

In correlation analysis, the level of 25(OH)D had an inverse relation with fasting glycemia (r = −0.7, *p <* 0.0001), postprandial blood glucose (r = −0.6, *p <* 0.0001), and HbA1c (r = −0.7, *p <* 0.0001), age (r = −0.4, *p <* 0.001), and BMI (r = −0.5, *p <* 0.0001), indicating that older patients with poor glycemic control and obesity are more likely to have lower values of 25(OH)D (Figure 1). The level of 25(OH)D was directly correlated with the diabetes duration (r = 0.1, *p =* 0.03), iron concentration (r = 0.2, *p =* 0.003), and eGFR (r = 0.2, *p =* 0.001). We applied univariate regression analysis to evaluate how a value of one parameter in a linear equation could predict the values of 25(OH)D. Figure 1 and Table 3 present the strength and direction of the inter-associations between studied variables.

To evaluate to what extent these potential factors are influencing the level of 25(OH)D (the dependent variable), taking into account the interaction between them, we performed a multivariate analysis, including in the first step all the factors, followed by successive eliminations and retesting in a backward-stepwise manner. The subtraction criterion of the predictor was an independent value of *p* > 0.1, and of the addition in the model, the *p*-value was <0.05. The most statistically significant model finally included only the HbA1c and patient’s age in an inverse relationship, with R^2^ adjusted 0.59 and a multiple correlation coefficient of 0.773 (*p <* 0.0001) (Table 4).

**Figure 2:**
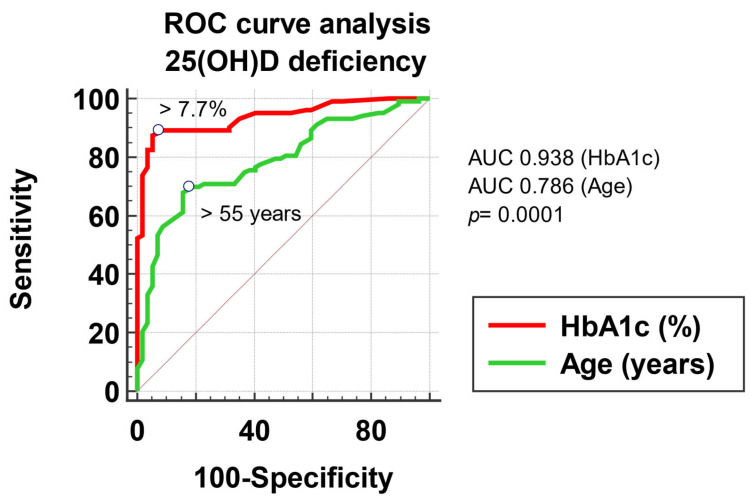
Graphical representation of the ROC curve analysis of 25(OH)D deficiency.

To evaluate the association of 25(OH)D deficiency with the factors included in the multivariate regression analysis, measured on a continuous scale (patient’s age and HbA1c), we built a multivariate logistic regression model, with potential predictors as of the variables mentioned above, respectively outcome, the 25(OH)D deficiency. We compared the ROC curves to evaluate the possibility of predicting a low 25(OH)D level based on the possible factors studied. According to the ROC curves, an age > 55 years and an HbA1c > 7.7% represent statistically significant predictive factors for 25(OH)D deficiency, with a sensitivity of 69.9 and specificity of 82.5 for age (AUROC 0.786, *p <* 0.001) and a sensitivity of 89.3 and a specificity of 92.9 for HbA1c (AUROC 0.938, *p <* 0.001) (Figure 2).

**Figure 3:**
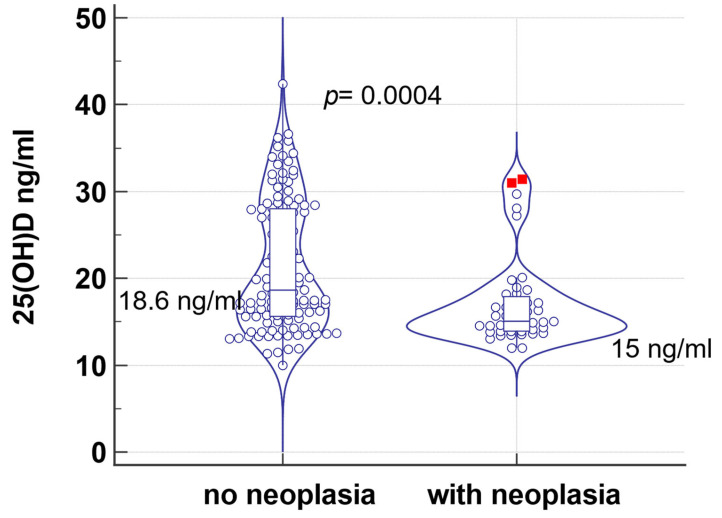
Comparison of 25(OH)D levels in patients with and without neoplasm.

In the present study, 26.8% (43/160) presented with different forms of neoplasia: 18.6% squamous-cellular carcinoma, 7% (3/43) gastric cancer, 14% (6/43) renal tumor, 9.3% (4/43) hepatocarcinoma, 9.3% (4/43) uterine cancer, 11.6% (5/43) pancreatic, 23.3% (10/43) prostatic adenocarcinomas, pharyngeal carcinoma 4.7% (2/43), and 2.3% (1/43) cerebral cancer. 25(OH)D levels were statistically significantly lower in patients with neoplasm than those without neoplasm: 15 ng/mL, compared to 18.6% ng/mL (*p* = 0.0004, Table 5, Figure 3). Moreover, patients with neoplasia were older (median age 74 vs. 47 years), with poorer glycemic control (median HbA1c 9.4% vs. 7.8%), higher body weight (median 92.5 kg vs. 81 kg) and BMI (median 31.5 vs. 28.8 kg/m^2^), with altered renal function (median eGFR 54 vs. 91 mL/min), anemia (median Hb 12.5 vs. 14.1 g/dL), and lower concentrations of iron (58 vs. 68.3 µg/dL) compared to the patients without neoplasia.

In the present study, the presence of cardiovascular disease was investigated, which included coronary heart disease, stroke, peripheral arterial disease, and aortic disease; 98 patients (61.2%) had previously documented CVD. Analyzing the 25(OH)D values according to CVD, we found that patients with T2DM and documented CVD had a statistically significant lower median of 25(OH)D compared to patients without CVD: 15.7 ng/mL vs. 20.9 ng/mL (*p* < 0.0001, Table 5).

**Figure 4:**
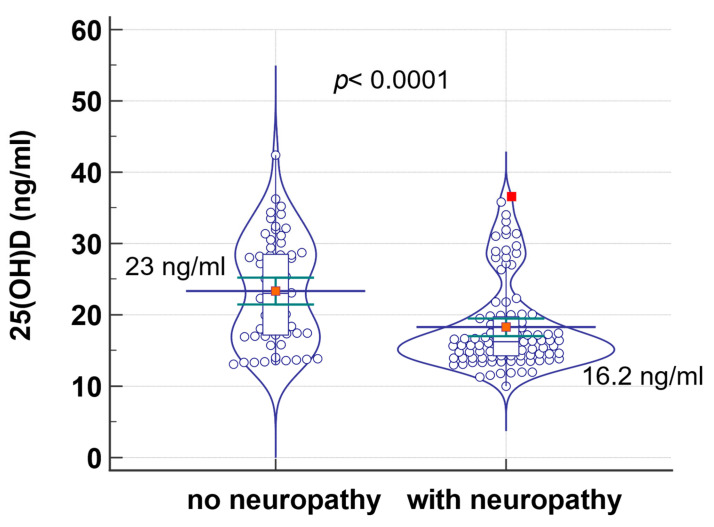
Comparison of 25(OH)D levels in patients with and without neuropathy.

Similarly, patients with diabetic neuropathy had statistically significantly lower 25(OH)D values than those without neuropathy: 16.2 ng/mL, compared to 23 ng/mL (*p* < 0.0001) (Table 5, Figure 4).

**Figure 5:**
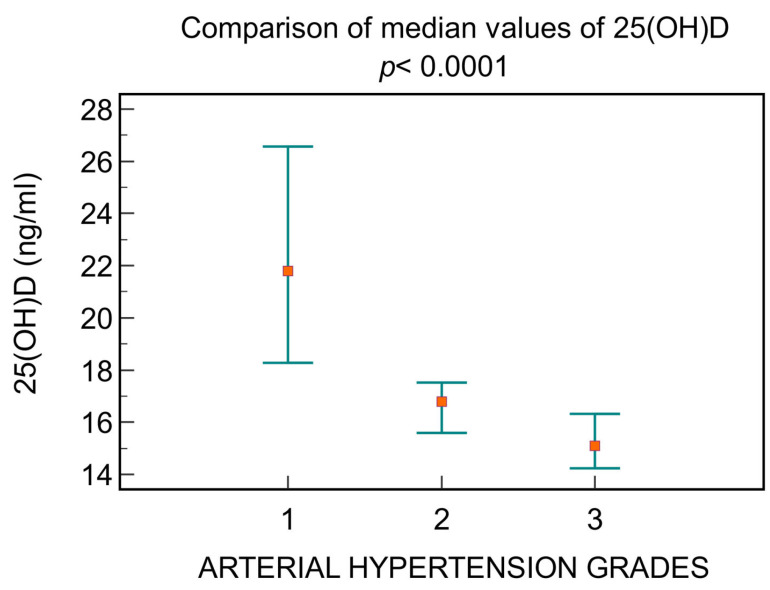
The differences between 25(OH)D levels depending on arterial hypertension grades.

Depending on the blood pressure, a decrease in the level of 25(OH)D is observed, directly correlated with increasing hypertension grades, as follows: patients with grade 1 had a median value of 25(OH)D 21.8 ng/mL [16.7; 28.4], those with grade 2 had 16.8 ng/mL [14.3; 20.1], and those with grade 3 had 15.09 ng/mL [13.5; 17] (*p <* 0.0001, Figure 5).

## 4. Discussion

The present study evaluated the levels of 25(OH)D in a Romanian cohort of patients with T2DM from Timisoara who were hospitalized between September 2023 and January 2024 for different medical reasons. They were assessed for vitamin D deficiency and its impact on their health. The results showed that these patients had a median level of 17.3 ng/mL 25(OH)D, with 63.8% having an important deficiency. About 30% of the patients presented related symptoms to 25(OH)D deficiency.

We found no differences between genders regarding vitamin D deficiency. However, the data are very heterogeneous in similar research. The 2001–2004 NHANES study did not reveal significant differences between the two sexes related to vitamin D deficiency, while another study from America showed that women have higher vitamin D levels than men [22,23]. In Africa, the prevalence of vitamin D deficiency was higher in women, while in the United Arab Emirates (UAE), it was similar in both sexes [24,25]. Our results show that the median was similar in men and women (17 ng/mL, compared to 18.2% ng/mL). Also, it appears that cultural and religious factors may influence vitamin D concentrations; thus, a higher prevalence of vitamin D deficiency among Muslim women was observed; for example, in Lebanon, Muslim women had lower serum vitamin D levels than Christian women [26]. We had no data about cultural and religious aspects in our study.

Numerous studies have shown the link between obesity and vitamin D levels, including in tropical countries [27,28,29,30]. A factor determining the inverse relationship between vitamin D deficiency and obesity is the reduced mobility of those with increased BMI, implicitly with less exposure to the sun [27]. A meta-analysis investigating the relationship between obesity and vitamin D demonstrated a higher prevalence of deficiency among obese subjects, independent of other risk factors, and obesity even reduced the effects of vitamin D supplementation [31,32]. The inverse correlation between body fat and vitamin D concentration may be due to the decreased bioavailability of vitamin D3 from dietary and skin sources due to the accumulation of vitamin D in body fat compartments, a situation observed even in elderly patients [33,34].

In Romania, suboptimal vitamin D levels are common (59%), especially in older persons and females. That is why, in our country, vitamin D supplementation is recommended from January to April [35]. Timisoara belongs to the moderate temperate continental climate, characteristic of the southeastern part of the Pannonian region, with some Mediterranean influences [36]. During the spring and summer, significant precipitation is recorded. Even during the winter, rain and snow often come from the Atlantic. We have 143 days/year with good temperatures, averaging above 15 degrees Celsius, between 7 May and 26 September [37].

Qu et al. analyzed the link between vitamin D deficiency and diabetic neuropathy. They demonstrated that Caucasian and Asian patients with vitamin D deficiency had a higher risk of diabetic neuropathy compared with those with normal vitamin D values [38]. Chair recommends vitamin D supplementation to prevent diabetic neuropathy in patients with T2DM. Luo et al. found similar results in a meta-analysis in which DM and vitamin D deficiency patients had a much higher risk of diabetic retinopathy [39]. In our group, patients with diabetic neuropathy had lower values of vitamin D compared to those without diabetic neuropathy: 16.2 ng/mL, compared to 23 ng/mL (*p* < 0.0001).

Numerous studies have demonstrated a direct relationship between all causes of mortality and vitamin D deficiency. Thus, Wan et al. showed that increased vitamin D levels are associated with a lower mortality rate among patients with T2DM. Another study by Lu et al. showed that among 15.195 adults, only 23% had sufficient vitamin D levels, and patients with vitamin D levels below 30 nmol/L had significantly higher all-cause mortality (*p* < 0.001) [40,41]. In our study, vitamin D deficiency was more severe in patients with neoplasia: 17 ng/mL, compared to 21.3% ng/mL (*p* = 0.0005).

Vitamin D decreases inflammatory markers (C-reactive protein, Interleukin 10) and oxidative stress markers (free radicals, nitric oxide), thus influencing atherosclerosis. The role of calcitriol in reducing renin synthesis was demonstrated, as well as its important role in the proliferation process of cardiomyocytes and vascular muscle cells [42,43,44,45].

Giovannuci et al. demonstrated, in the Health Professionals Follow-up Study, which included 18,225 men, that the risk of myocardial infarction is 2.4 times higher in those with a vitamin D level < 15 ng/mL compared to those with values > 30 ng/mL [46].

In the Framingham Offspring Study, over 5.4 years, hypertensive patients with a serum level of 25(OH)D < 15 ng/mL were 53% more likely to have a cardiovascular event compared to those with a serum level of 25(OH)D greater than 15 ng/mL. Patients with values < 10 ng/mL were 80% more likely to have a cardiovascular event [47]. Similarly, in our study that included patients with DM, those with CVD had a statistically significantly lower median of vitamin D than those without CVD: 15.7 ng/mL, vs. 20.9 ng/mL (*p* < 0.0001).

Our logistic regression model indicates that age and glycemic control (fasting glycemia, postprandial glycemia, and HbA1c) are the strongest predictors of the risk of low vitamin D values. Vitamin D deficiency is also correlated with other factors, such as DM duration, iron concentration, and renal function assessed by eGFR. A recent systematic review and meta-analysis of 46 randomized clinical trials showed that vitamin D supplementation reduced the HbA1c up to 0.2%, the beneficial effect on glycemic control being more intense in T2DM patients with 25(OH)D deficiency [48]. The relationship between age and lower values of vitamin D may be explained by the reduced mobility of the elderly and, therefore, their inability to be exposed to the sun.

The present study could not establish possible mechanisms underlying the associations between the reduced levels of 25(OH)D and studied comorbidities like diabetic neuropathy, CVD, stroke, HTN, neoplasia, dementia, or poor glycemic control. However, important evidence in the literature links vitamin D to the β-cell function, explained by the presence of the VDR in β-cells and the vitamin D-dependent calcium-binding proteins in the pancreas [49,50]. Studies on in vitro and in vivo models have proved that vitamin D is essential for normal insulin release and glucose tolerance [51,52,53,54]. Some studies have suggested that vitamin D deficiency may play a role in the development and progression of diabetic neuropathy, as well as its symptoms. Vitamin D may increase insulin secretion and sensitivity and improve β-cell function, thus reducing hyperglycemia and oxidative stress, major nerve damage factors [55]. Vitamin D may modulate the expression of neurotrophic factors, such as nerve growth factor and glial cell line-derived neurotrophic factor, which are essential for nerve survival and regeneration. Also, vitamin D may inhibit the production of pro-inflammatory cytokines, such as tumor necrosis factor-alpha and interleukin-6, which are involved in nerve inflammation and degeneration [56].

Moreover, vitamin D may enhance the function of calcium channels, which are important for nerve conduction and neurotransmission [57]. Regarding diabetic macroangiopathy, vitamin D is involved in reducing chronic inflammation and oxidative stress, which are major factors in endothelial dysfunction, atherosclerosis, and modulating angiogenesis, which is impaired in diabetes. Consequently, the healing of vascular injuries is altered [58].

For more than 25 years, there has been ample evidence of the regulatory role of vitamin D in decreasing blood pressure by inhibiting the renin–angiotensin–aldosterone system activity [59] through reduced gene expression [60].

Over the past years, researchers have demonstrated that vitamin D and its active metabolites are useful in preventing or treating various forms of cancers through many mechanisms, such as gene transcription regulation, apoptosis of tumoral cells, DNA repair, antioxidant protection, and immunomodulation [61].

Vitamin D plays a crucial role in dementia prevention and management. One of the mechanisms confirming this hypothesis is that it upregulates the production of several neurotrophic factors, which promote the survival, development, and function of neurons [62,63]. Furthermore, in animal studies, chronic supplementation with vitamin D appears to protect against the neurotoxicity of glutamate exposure [64].

Also, the study did not evaluate the impact of different concomitant medications on 25(OH)D levels. Further large-scale research is needed to investigate the possible pleiotropic effects of vitamin D supplementation for preventing deficiency and whether optimal vitamin D levels would prevent chronic complications of T2DM, respectively, and other comorbidities often found in these patients.

## 5. Conclusions

Vitamin D deficiency is associated with several comorbidities that reduce the quality of life. It is useful for this blood test to become routine in clinical practice both in the case of patients with DM already diagnosed as well as those at risk (overweight, obese, hypertensive, with dyslipidemia, nonalcoholic steatohepatitis, and metabolic syndrome). T2DM patients with age > 55 years and HbA1c > 7.7% are more likely to have insufficient levels of vitamin D. Thus, these subgroups are suitable for appropriate supplementation with vitamin D accompanied by a healthy lifestyle and diet.

## Figures and Tables

**Table 1:** Comparison of general characteristics of the studied patients by gender.

Number	Total	Women	Men	*p*-Value *
% (number) ^c^	100% (160)	49.3 (79)	50.7 (81)	0.8
Age (years) ^b^	57 [37; 69]	55	61	0.4
T2DM duration (years) ^b^	9.5 [5; 15]	9	10	0.8
Weight (kg) ^a^	86.1 ± 17.5	77.9 ± 15.8	94 ± 15.2	<0.0001 **
BMI (kg/m^2^) ^b^	30.2 [24.7; 34]	28.8	31.31	0.001 **
HbA1c (%) ^b^	8.3 [7.2; 8.6]	7.9	8.5	0.3
FG (mg/dL) ^b^	172 [142.5; 201]	168	176	0.2
PPG (mg/dL) ^b^	197 [166; 216]	194	197	0.1
Hb (g/dL) ^b^	14,1 [12.5; 14.8]	13.5	14.40	<0.0001 **
Iron (µg/dL) ^b^	65.9 [52.6; 88.1]	68.2	65	0.6
TSH (mU/L) ^b^	2 [1.3; 2.9]	2	2	0.9
eGFR (mL/min) ^b^	83 [54.5; 103]	72	87	0.03 **
25(OH)D ^b^	17.3 [14.5; 27]	18.2	17	0.4

^a^ T-test; ^b^ Mann–Whitney test, ^c^ Chi-squared test; * *p*-value for comparison of variables according to gender; ** *p*-value < 0.05 statistically significant; T2DM = type 2 diabetes mellitus; BMI = body mass index; HbA1c = glycated hemoglobin; FG = Fasting glycemia; PPG = Postprandial glycemia; Hb = hemoglobin; TSH = thyroid stimulating hormone; eGFR = estimated glomerular filtration rate; 25(OH)D = 25-hydroxy vitamin D.

**Table 2:** Comparison of metabolic and anthropometric markers in T2DM patients by 25(OH)D level.

Variable	Optimal 25 (OH)D		25(OH)D Insufficiency		25(OH)D Deficiency		*p* ^a^
	Median	Average Rank	Median	Average Rank	Median	Average Rank	
Age (years)	40	47	41	53.1	65.5	97.3	<0.0001
DM duration (years)	13	97.8	9	84.2	9	75.5	0.1
Weight (kg)	70	42.7	73	51.9	94.8	98.6	<0.0001
BMI (kg/m^2^)	24	37.9	24.7	50.3	30.1	100.1	<0.0001
HbA1c (%)	7	32.9	7.1	37.5	9.2	105.8	<0.0001
FG (mg/dL)	142	35.1	135	37.5	192	105.4	<0.0001
PPG (mg/dL)	160	43	161	42.2	210	102	<0.0001
eGFR (mL/min)	98	101	94	99.3	71	69.4	0.0003
Hb (g/dL)	14.1	94.8	14.2	95.9	13.5	71.9	0.008
Iron (µg/dL)	74.1	93.7	72.6	96.7	63.3	71.8	0.007
TSH (mU/L)	1.6	82.4	2.3	86.6	1.9	77.8	0.6

^a^ Kruskal–Wallis test; *p*-value < 0.05 statistically significant; T2DM = type 2 diabetes mellitus; BMI = body mass index; HbA1c = glycated hemoglobin; FG = fasting glycemia; PPG = postprandial glycemia; Hb = hemoglobin; TSH = thyroid stimulating hormone; eGFR = estimated glomerular filtration rate; 25(OH)D = 25-hydroxy vitamin D.

**Table 3:** Univariate regression analysis between 25(OH)D and the studied parameters.

Parameter	Coefficient	Standard Error	Coefficient of Determination R^2^	t	*p*
Age (years)	−0.16	0.02	0.19	−6.19	<0.0001
T2DM duration (years)	0.15	0.07	0.02	2.13	0.0339
FG (mg/dL)	−0.10	0.00	0.53	−13.52	<0.0001
PPG (mg/dL)	−0.10	0.00	0.39	−10.16	<0.0001
HbA1c (%)	−3.08	0.21	0.57	−14.47	<0.0001
Weight (kg)	−0.21	0.02	0.29	−8.06	<0.0001
BMI (kg/m^2^)	−0.74	0.08	0.34	−9.13	<0.0001
Hb (g/dL)	0.92	0.34	0.04	2.68	0.0080
Iron (µg/dL)	0.05	0.02	0.05	2.99	0.0032
eGFR (mL/min)	0.06	0.01	0.06	3.31	0.0011

Abbreviations: T2DM = type 2 diabetes mellitus; FG = fasting glycemia; PPG = postprandial glycemia; HbA1c = glycated hemoglobin; BMI = body mass index; Hb = hemoglobin; eGFR = estimated glomerular filtration rate; *p <* 0.05 statistically significant.

**Table 4:** Multivariate regression analysis between 25(OH)D and the studied parameters.

Independent Variables	Coefficient	Standard Error	t	*p*	r_partial_	r_semipartial_
(Constant)	48.0796					
HbA1c	−2.8022	0.2234	−12.5	<0.0001	−0.7	0.6347
Age	−0.06831	0.02069	−3.3	0.001	−0.2	0.1670

HbA1c = glycated hemoglobin.

**Table 5:** The median values of 25(OH)D in patients with and without different comorbidities.

Comorbidity	With Disease			Without Disease			
	*n*	Median	Average Rank	*n*	Median	Average Rank	*p* ^a^
Neoplasia *	43	15	59.2	117	18.6	88.3	0.0004
Diabetic neuropathy *	99	16.2400	68.0253	61	23.0000	100.7459	<0.0001
CVD *	74	15.7	60.8	86	20.9	60.8	<0.0001
Dementia *	29	15.7	60.2	131	18.2	84.9	0.009
Stroke *	33	16.2	62.9	127	18.3	85	0.01

^a^ Mann–Whitney test. * Statistical test used.

## Data Availability

Due to the retrospective study design, patients included in this study did not give written consent for their data to be shared publicly, so due to the sensitive nature of the research, supporting data are not available.
